# Identification of TRPM2 as a prognostic factor correlated with immune infiltration in ovarian cancer

**DOI:** 10.1186/s13048-023-01225-y

**Published:** 2023-08-22

**Authors:** Wei Huang, Yuliang Wu, Ning Luo, Xueqian Shuai, Jing Guo, Chunyan Wang, Fanchun Yang, Li Liu, Shupeng Liu, Zhongping Cheng

**Affiliations:** 1 Department of Obstetrics and Gynecology, Shanghai Tenth People’s Hospital, Tongji University School of Medicine, 301 Yanchang Road, Shanghai, 200072 China; 2 Gynecologic Minimally Invasive Surgery Research Center, Tongji University School of Medicine, 1239 Siping Road, Shanghai, 200092 China

**Keywords:** Pan-cancer, TCGA, TRPM2, Prognosis, Immunology, Pyroptosis

## Abstract

**Introduction:**

Ovarian cancer (OC) is one of the most common gynecologic malignant cancers with the current survival rate remaining low. TRPM2 has been reported as a survival predictor in various cancers but not in OC. The aim of this study is to explore the role and its underlying mechanism of TRPM2 in OC.

**Methods:**

The transcriptome data and clinical data were obtained from TCGA, GTEx, and GEO (GSE17260). DriverDBv3 and PrognoScan were used to analyze survival correlations. GSEA analysis was performed to uncover the underlying mechanism. The correlations between TRPM2 and immune score, immune cell infiltration were analyzed by TIMER2.0.

**Results:**

TRPM2 was highly expressed in OC and high TRPM2 expression was related to the poor prognosis based on the Kaplan-Meier curves, univariate and multivariate analysis. The enrichment analysis suggested that TRPM2 was involved in immune-related pathways. Positive correlations were also observed between TRPM2 expression and immune score and immune cells covering B cells, T cells, macrophage, neutrophil, and myeloid dendritic cells. We also found that TRPM2 was positively related to immune checkpoints including ICOSLG, CD40, CD86, etc. TRPM2 expression had a positive correlation with M2 macrophage, but not with M1 macrophage. Besides, TRPM2 showed a strong positive correlation with pyroptosis-related genes including NLRP3, NLRC4, NOD2, NOD1, IL1B, GSDMD.

**Conclusion:**

Our study demonstrated that TRPM2 is a poor prognostic prediction factor in ovarian cancer and is correlated to the immune microenvironment and pyroptosis. TRPM2 may act as a new immunotherapy target, which promoted the survival rate of OC patients.

**Supplementary Information:**

The online version contains supplementary material available at 10.1186/s13048-023-01225-y.

## Introduction

Ovarian cancer (OC) is one of the most common gynecologic malignant cancers in the world, with most of the patients diagnosed at an advanced stage and having poor outcomes [1]. It is the seventh most diagnosed cancer among women and the five-year survival rate is 46% [2]. Currently, the treatment for OC containing surgery, chemotherapy, immunotherapy, and targeted therapy such as Poly (ADP-ribose) polymerase (PARP) inhibitors, have improved the prognosis of OC patients. However, the survival rate remains low due to the complex molecular mechanisms and tumor microenvironment [3].

The transient receptor potential (TRP) is superfamily of ion channels involved in the modulation of physiological function and tumor progression [4]. TRP family contains TRPC, TRPV, TRPM, TRPP, TRPML, TRPA, and TRPN [5]. Among these, TRPM2 is a non-selective cationic channel, consisting of an approximate 800 amino acid N-terminal area, a C-terminal coiled-coil loop, three extracellular loops root in six transmembrane domains (S1-6), and ADP-ribose (ADPR). The N- and C-terminals of TRPM2 are located in intracellular, and the pore-forming loop is situated on S5 and S6 [6]. TRPM2 has been proved to increase the concentration of intracellular Ca^+ 2^, which provides positive feedback for TRPM2 activation [7]. Additionally, TRPM2 also plays an important role in the reactive oxygen species (ROS) pathway in physiological and pathological activities [8, 9]. Early research illustrated that TRPM2 promoted cell apoptosis in the endothelial or neuronal cells and responded to oxidative stress in male-specific ischemic injury by modulating Ca^+ 2^ [10]. Nowadays, some reports demonstrate that TRPM2 is correlated to innate immunity regulation and inflammation, resulting from the presence of TRPM2 on the cells of monocytic lineage, lymphocytes, and neutrophils [11–13].

TRPM2 is also found to be involved in tumor development. Latest studies reported that TRPM2 was highly expressed on various cancer including bladder, breast, head and neck, lung, pancreatic, prostate, neuroblastoma, which might hint that TRPM2 could promote tumor progression [5, 14]. TRPM2 could sustain tumor cell viability by activating transcription factors such as hypoxia-inducible factor-alpha (HIF-1/2α), cAMP-responsive element-binding protein (CREB), and nuclear factor (erythroid-derived 2)-related factor-2 (Nrf2), subsequently modulating the downstream pathways including mitochondrial function maintenance, ATP production, cell autophagy, DNA repair, cellular bioenergetics, and ROS production [15–20]. Inhibition of TRPM2 could cause tumor cell death, and promote the tumor drug sensitivity in T cell leukemia, gastric cancer, breast cancer cells, and neuroblastoma, to chemotherapeutic agents [16–19, 21–23]. TRPM2 inhibition also leads to increased DNA damage and cytotoxicity in triple-negative or estrogen-receptor-positive breast cancer, reducing survival and restrain migration of tongue carcinoma, and enhancing the radiation sensitivity in T-cell leukemia [14]. Thus, TRPM2 is a potential target for anti-cancer therapy in various tumors. However, the role and underlying mechanisms of TRPM2 in OC are still unclear.

In the present study, we found that TRPM2 was significantly highly expressed in most tumors including OC based on The Cancer Genome Atlas (TCGA), Genotype-Tissue Expression (GTEx). Survival analysis indicated that high expression of TRPM2 predicted poor survival in OC. Subsequent bioinformatics analyses found that TRPM2 expression was positively correlated with immune-associated pathway, immune score, majority immune checkpoints, and pyroptosis. These results suggested that TRPM2 could be a poor prognostic predictor in OC, which was possibly modulated by immune response suggested by the association between TRPM2 expression and the tumor immune microenvironment.

## Results

### The prognostic value of TRPM2 in pan-cancer and ovarian cancer

**Fig. 1:**
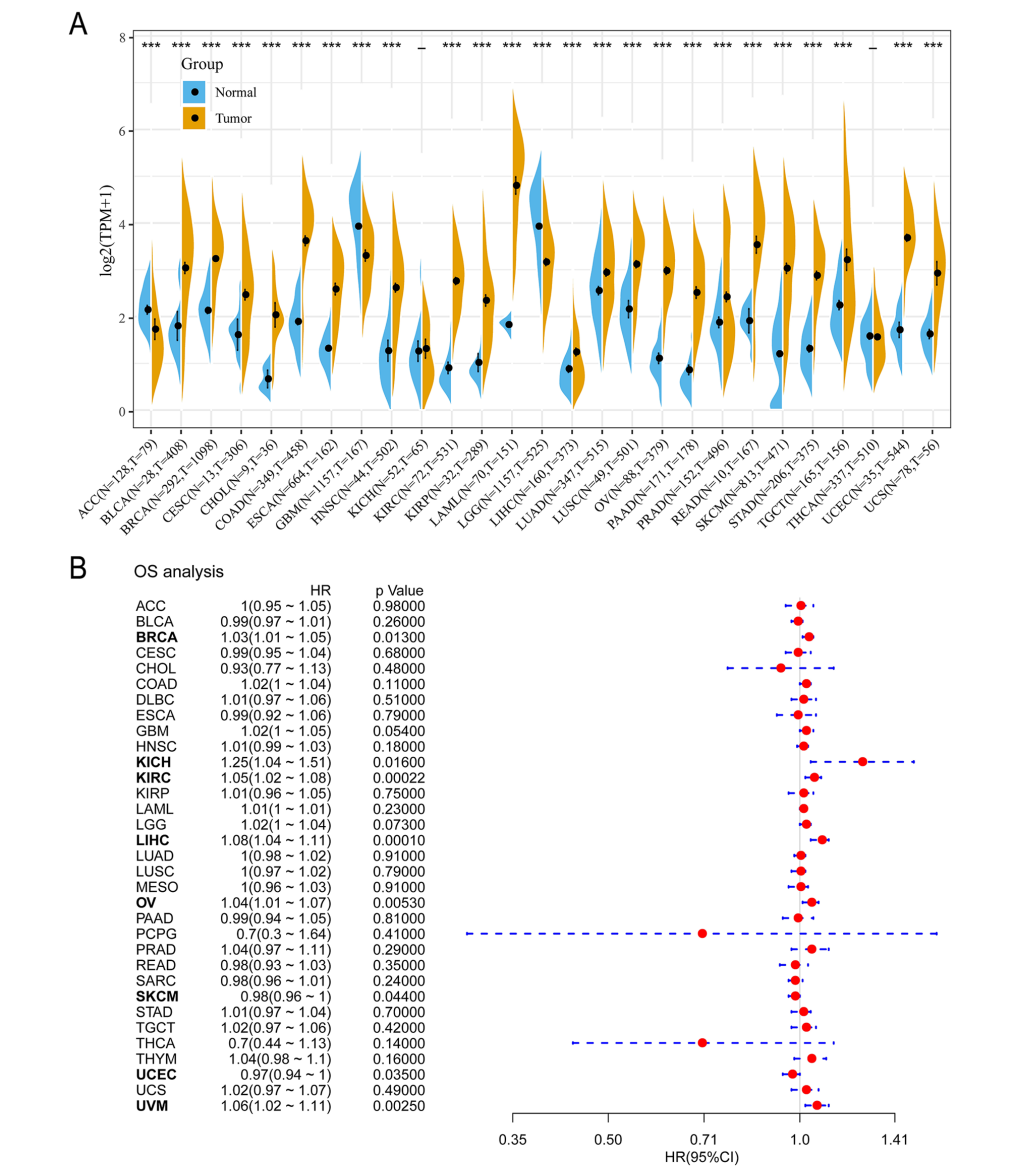
Aberrantly expression and prognosis value of TRPM2 in human pan-cancer. (**A**) TRPM2 expression between tumor tissue and normal tissue based on TCGA database and GTEx database. (**B**) Correlation between TRPM2 expression and prognostic value analyzed by the Univariate Cox proportional hazards regression model. (p < 0.05 was considered significant, *p < 0.05, **p < 0.01, ***p < 0.001.)

To explore the expression pattern of TRPM2 in multiple tumors, we firstly evaluated the expression of TRPM2 mRNA in pan-cancer and normal tissues based on TCGA and GTEx databases. The result showed that TRPM2 was upregulated in most tumors including bladder urothelial carcinoma (BLCA), breast invasive carcinoma (BRCA), cervical squamous cell carcinoma (CESC), Cholangiocarcinoma (CHOL), colon adenocarcinoma (COAD), esophageal carcinoma (ESCA), head and neck squamous cell carcinoma (HNSC), kidney renal clear cell carcinoma (KIRC), kidney renal papillary cell carcinoma (KIRP), acute myeloid leukemia (LAML), liver hepatocellular (LIHC), lung adenocarcinoma (LUAD), lung squamous cell carcinoma (LUSC), ovarian serous cystadenocarcinoma (OV), pancreatic adenocarcinoma (PAAD), Prostate adenocarcinoma(PRAD), Rectum adenocarcinoma (READ), skin cutaneous melanoma (SKCM), stomach adenocarcinoma (STAD), testicular germ cell tumors (TGCT), uterine corpus endometrial carcinoma (UCEC), and uterine carcinosarcoma (UCS) (Fig. 1A). And TRPM2 was downregulated in adrenocortical carcinoma (ACC), glioblastoma multiforme (GBM), and brain lower-grade glioma (LGG) (Fig. 1A). No significant alteration of TRPM2 mRNA expression was observed in Kidney Chromophobe (KICH) and Thyroid carcinoma (THCA).

Subsequently, we analyzed the correlation between TRPM2 expression and prognosis of patients with the univariate Cox proportional hazards regression model. Overall survival (OS), disease-specific survival (DSS), disease-free interval (DFI), and progression-free interval (PFI) analyses were performed based on TCGA database. It demonstrated that TRPM2 was a hazard factor of OS in tumors such as BRCA, KICH, KIRC, LIHC, OV, UCEC, and UVM, while it was protective factor of OS in SKCM, UCEC (Fig. 1B). Meanwhile, the survival analyses also suggested that TRPM2 expression had impacted BRCA in DSS, BRCA, COAD, GBM, KICH, KIRC, LIHC, OV, UVM in PFI, and BRCA, GBM, KICH, KIRC, LIHC, THYM, UVM in DFI. (Supplementary Fig. 1A-C).

Based on the observation that TRPM2 was a hazard factor for ovarian cancer in OS and PFI analyses, we further verified the association with the Univariate and Multivariate Cox proportional hazards regression model analyses based on PrognoScan (http://dna00.bio.kyutech.ac.jp/PrognoScan/index.html) [24]. The univariate analyses result showed that TRPM2, as well as age and TNM stage, were risk factors, and the multivariate analyses results showed that age, TNM stage, TRPM2, race were risk factors (Table 1).

**Table 1:** Univariate and multivariate analyses for ovarian cancer on clinicopathological characteristics

Clinical characteristic	Univariate analysis				Multivariate analysis			Uni/Multi Prognostic
	p-value	HR	95%CI		p-value	HR	95%CI	
Age(years)	**< 0.0001**	1.56	1.26–1.94		**0.0089**	1.44	1.10–1.90	Poor/Poor
TRPM2	**0.0183**	1.37	1.05–1.78		**0.028**	1.37	1.03–1.81	Poor/Poor
Grade	0.168	1.23	0.92–1.64		0.7002	1.08	0.72–1.64	-/-
Race	0.0986	0.81	0.63–1.04		**0.0082**	0.67	0.47–0.90	-/Good
TNM stage	**0.0008**	1.43	1.16–1.76		**0.0244**	1.43	1.05–1.94	Poor/Poor

HR: Hazard Ratio; CI: confidenceinterval; TNM: T, tumor; N, node; M, metastasis. *p < 0.05, **p < 0.01, ***p < 0.001, ****p < 0.0001.

**Fig. 2:**
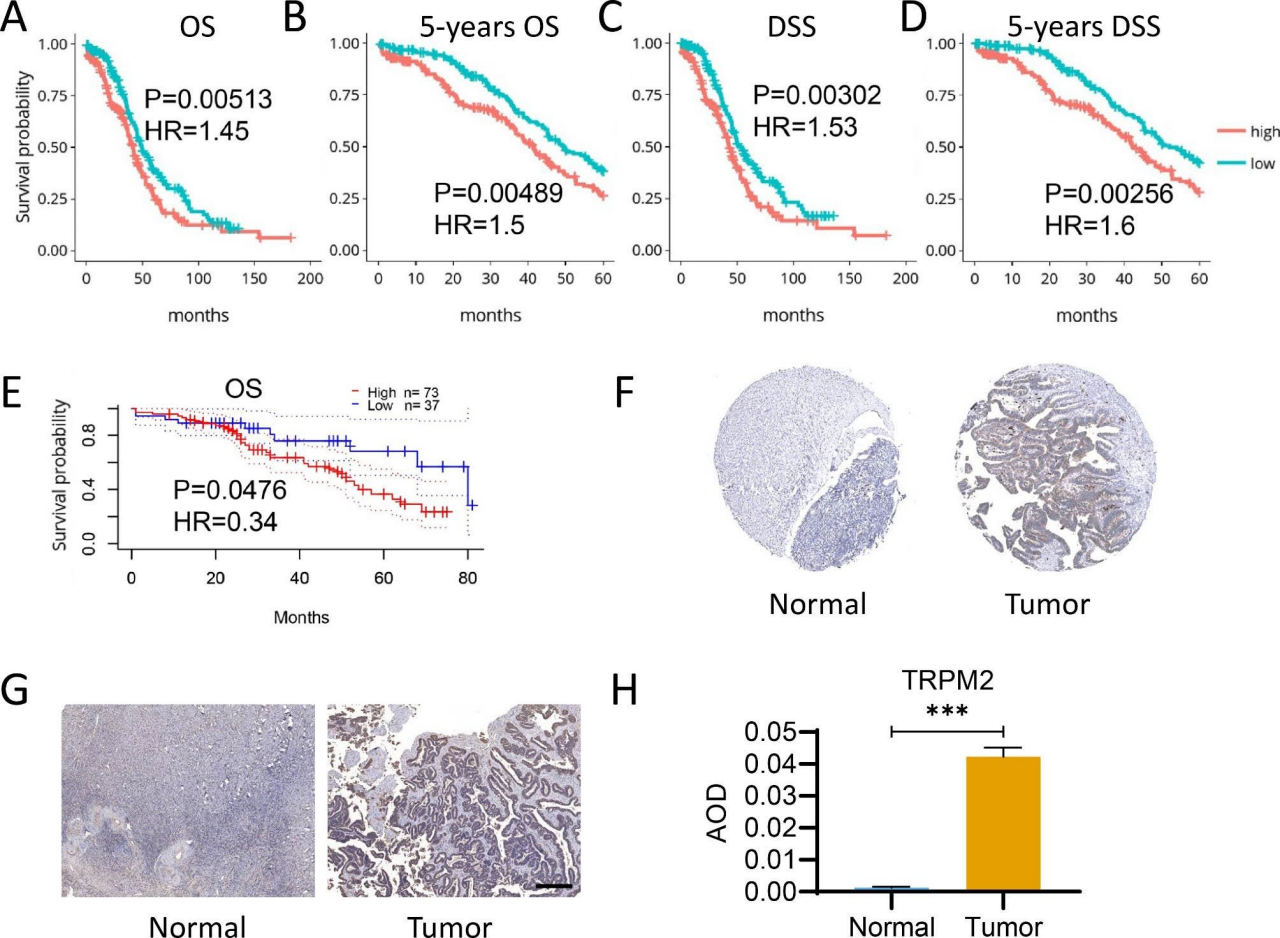
The prognostic feature of TRPM2 expression in ovarian cancer according to DriverDBv3 and PrognoScan. (**A-D**) Association between TRPM2 expression and OS (**A**), 5-years OS (**B**), DSS (**C**), and 5-years DSS (**D**) in ovarian cancer based on DriverDBv3. **(E)** Association between TRPM2 expression and OS in ovarian cancer in PrognoScan. (p < 0.05 was considered significant. n: TRPM2-high = 186,TRPM2-low = 186) **(F)** Immunohistochemistry result of TRPM2 expression in ovarian cancer tissuse (Tumor) and normal tissue (Normal) according to The Human Protein Atla. **(G-H)** Immunohistochemistry result of TRPM2 expression **(G)** and average optical density (AOD) analysis of clinical ovarian cancer tissue samples (Tumor) and normal tissue sample (Normal). N = 5, Student’s t-test, ***p < 0.001. (Scale bar = 400 μm, n = 5)

Kaplan-Meier survival estimate analysis was subsequently performed to further explore the association of TRPM2 expression and survival in TCGA-OV [25]. Notably, Kaplan-Meier plots showed that high TRPM2 expression was correlated to poor prognosis in OS, 5-year OS, DSS, and 5-year DSS (Fig. 2A-D). Accordingly, the correlation was also observed in the GSE17260 dataset (Fig. 2E). Immunohistochemistry results in The Human Protein Atlas suggested a high expression of TRPM2 in ovarian cancer tissues compared with normal tissues (Fig. 2F), which was also confirmed by our clinical samples (Fig. 2G-H), showing that TRPM2 is highly expressed in ovarian cancer patients. The results indicated that TRPM2 expression was significantly related to the prognosis of ovarian cancer patients and may act as a risk factor in OC.

### The function enrichments analysis based on TRPM2 expression in ovarian cancer

**Fig. 3:**
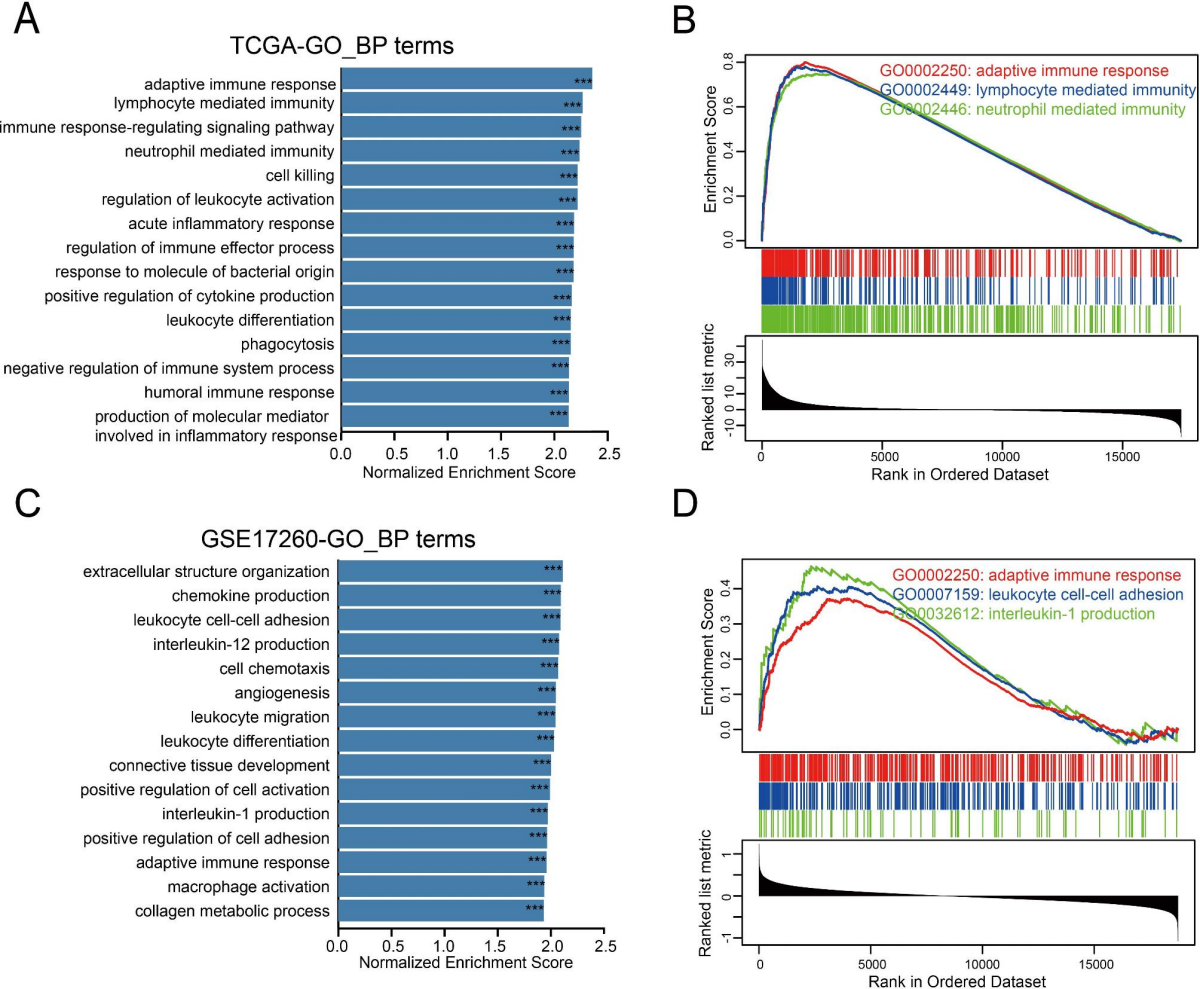
Gene Set Enrichment Analysis (GSEA) of TRPM2 in TCGA-OV and GSE17260 dataset. **(A)** GO functional enrichment analysis of biological process in TCGA-OV. **(B)** Biological process of immune-related pathways were in TCGA-OV. **(C)** GO functional enrichment analysis of biological process in GSE17260. **(D)** Biological process of immune-related pathways were in GSE17260. (***p < 2.2e-16)

To clarify the underlying mechanisms of TRPM2 affecting the survival of ovarian cancer patients, we conducted a GSEA algorithm with TCGA-OV and GSE17260 databases after the calculation of fold change of genes according to TRPM2 expression. GO enrichment analysis of biological process showed that high TRPM2 expression was positively correlated with immune-related pathways such as adaptive immune response, lymphocyte mediated immunity, and neutrophil mediated immunity (Fig. 3A-B) on TGCA-OV dataset. Similarly, the results emphasized that TRPM2 expression was positively related to immune-related pathways including interleukin-1 production, leukocyte cell-cell adhesion, and adaptive immune response on the GSE17260 dataset (Fig. 3C-D). Additionally, KEGG pathways further illustrated that TRPM2 was positively related to immune-related pathways covering Th1 and Th2 cell differentiation, cell adhesion molecules (CAMs), Th17 cell differentiation, and Natural killer cell mediated cytotoxicity (Supplementary Fig. 2A-D).

### Correlation between TRPM2 expression and the immune microenvironment in ovarian cancer

**Fig. 4:**
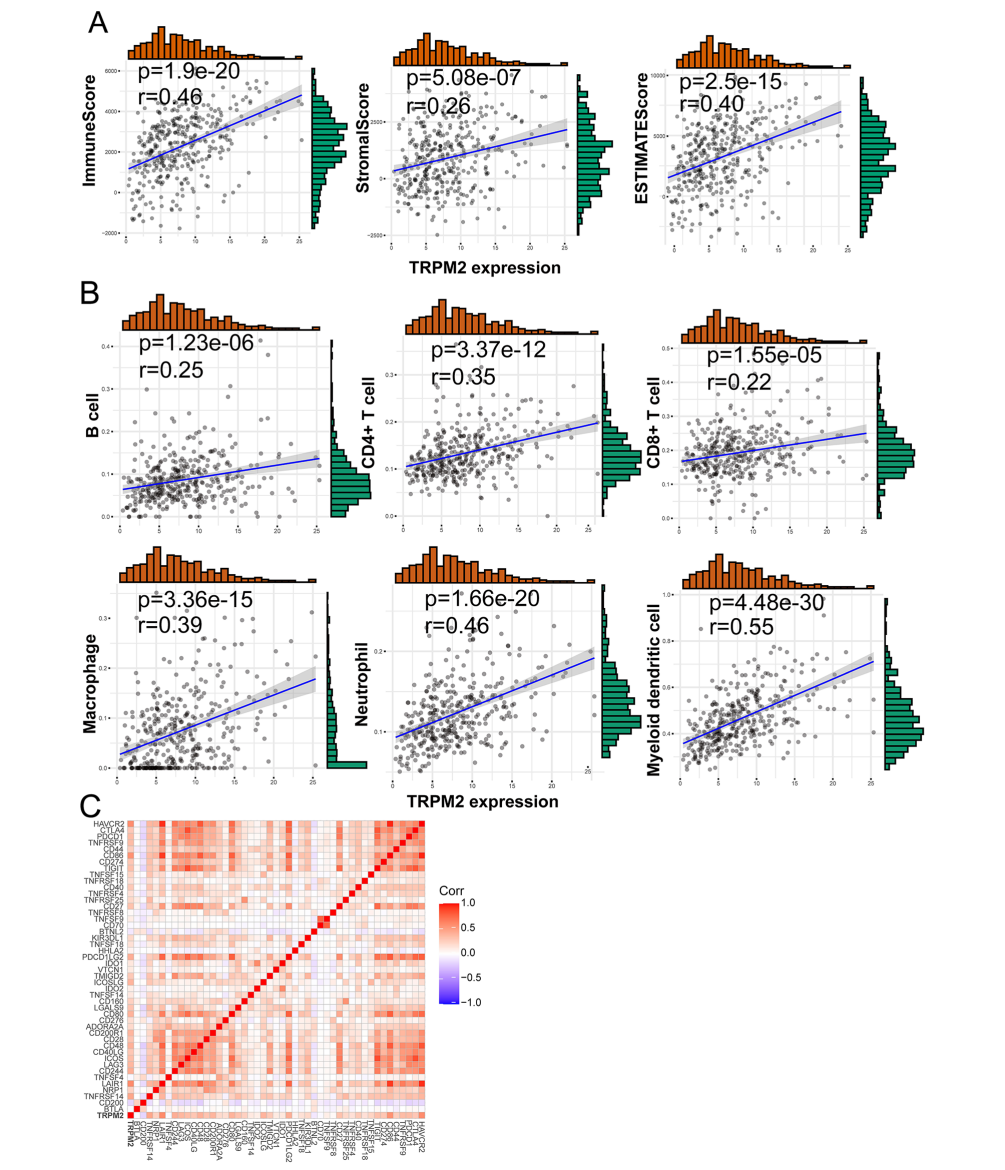
Correlations between TRPM2 expression and ImmuneScore, StromalScore, ESTIMATEScore, immune cell infiltrations and immune checkpoints in ovarian cancer. **(A)** Correlation between TRPM2 expression and ImmuneScore, StromalScore and ESTIMATEScore. **(B)** Correlation between TRPM2 expression and B cell, CD4^+^ T cell, CD8^+^ T cell, Macrophage, Neutrophil, and Myeloid dendritic cell. **(C)** Correlation between TRPM2 expression and immune checkpoints. (p < 0.05 was considered significant and all data comes from TCGA-OV database.)

As TRPM2 was shown to be associated with immune-related pathways, we investigated the correlation between TRPM2 expression and immune microenvironment including immune score, immune cell infiltration, and immune checkpoints based on TCGA-OV dataset. The immune score was evaluated by ESTIMATE analysis and a positive correlation between TRPM2 expression and immune score, stromal score, and estimate score in TCGA-OV was observed (Fig. 4A). Further, we evaluated the correlation between TRPM2 and tumor-infiltrating immune cells including B cell, CD4^+^ T cell, CD8^+^ T cell, macrophage, neutrophil, and myeloid dendritic cells. The results suggested that TRPM2 expression was appreciably positively correlated with these immune cells based on TIME2.0 (Fig. 4B). Among the correlated cells, innate immune cells including macrophage, neutrophil, and myeloid dendritic cells were more related to the expression of TRPM2. The analysis using Cibersort and quanTIseq also suggested TRPM2 was positively correlated to macrophage, neutrophil, and dendritic cells (Supplementary Fig. 3A-B).

Currently, immune checkpoint inhibitors, as a novel cancer therapy, play a vital role in tumor immunotherapy. Thus, we subsequently analyzed the relationship between TRPM2 expression and 46 immune checkpoints. The result showed that TRPM2 expression was significantly correlated with 21 immune checkpoints including ICOSLG, TNFRSF18, CTLA4, CD86, TNFRSF4, CD40, TIGIT, LAIR1, CD48, TNFRSF14, CD27, LGALS9, HAVCR2, KIR3DL1, TMIGD2, CD40LG, ICOS, IDO2, IDO1, PDCD1LG2, CD80 (Fig. 4C). To sum up, these results suggested that TRPM2 might act as an important role in the regulation of the immune microenvironment in ovarian cancer.

### Correlation between TRPM2 expression and specific immune cell in ovarian cancer

To further evaluate the relationship between TRPM2 and tumor immunity, we analyzed the correlation between TRPM2 expression and immune cell markers based on TCGA-OV dataset. Our result illuminated that TRPM2 was a closely related factor to most immune cells based on the correlation analyses of immune cell markers, particularly to the T cell, B cell, monocyte, tumor-associated macrophage, M2 macrophage, dendritic cell, Th1 cell, Th2 cell, Tfh cell, Treg cell, and T cell exhaustion (Table 2).

**Table 2:** Correlationship between the TRPM2 expression and gene markers of immnue cells in TCGA-OV.

Description	Gene maker	Cor	p-value
CD8 + T cell	CD8A	0.36	6.27E-13
	CD8B	0.00	9.49E-01
T cell	CD3D	0.29	1.87E-08
	CD3E	0.36	1.25E-12
	CD2	0.38	3.92E-14
B ell	CD19	0.12	1.89E-02
	CD79A	0.13	1.43E-02
Monocyte	CD86	0.56	9.87E-32
	CD115 (CSF1R)	0.61	8.69E-39
TAM	CCL2	0.12	2.60E-02
	CD68	0.35	4.81E-12
	IL10	0.21	6.60E-05
M1 Macrophage	INOS (NOS2)	0.01	9.18E-01
	IRF5	0.42	4.61E-17
	COX2 (PTGS2)	0.10	5.52E-02
M2 Macrophage	CD163	0.51	1.00E-25
	VSIG4	0.49	9.35E-24
	MS4A4A	0.39	5.54E-15
Neutrophils	CD66 b (CEACAM8)	-0.04	4.54E-01
	CD11b (ITGAM)	0.59	2.99E-36
	CCR7	0.26	4.46E-07
Natural killer cell	KIR2DL1	0.16	2.48E-03
	KIR2DL3	0.08	1.42E-01
	KIR2DL4	0.24	2.46E-06
	KIR3DL1	0.21	6.91E-05
	KIR3DL2	0.06	2.39E-01
	KIR3DL3	0.22	2.68E-05
Dendritic cell	HLA- DPB1	0.35	3.13E-12
	HLA- DQB1	0.30	5.18E-09
	HLA- DRA	0.32	5.11E-10
	HLA- DPA1	0.39	7.36E-15
	BCDA-1 (CD1C)	0.21	3.69E-05
	BDCA-4 (NRP1)	0.24	3.14E-06
	CD11c (ITGAX)	0.57	3.71E-33
Th1	T-bet (TBX21)	0.44	5.87E-19
	STAT4	0.25	1.60E-06
	STAT1	0.25	1.09E-06
	IFN-γ(IFNG)	0.25	6.87E-07
	TNF-α (TNF)	0.08	1.27E-01
Th2	GATA3	0.04	4.26E-01
	STAT6	0.41	2.65E-16
	STAT5A	0.45	6.23E-20
	IL-13	0.14	6.88E-03
Tfh	BCL6	0.20	9.41E-05
	IL-21	0.17	1.16E-03
Th17	STAT3	0.34	9.94E-12
	IL17A	0.02	7.09E-01
Treg	FOXP3	0.42	5.26E-17
	CCR8	0.07	1.80E-01
	STAT5B	0.17	1.22E-03
	TGFβ (TGFB1)	0.43	5.85E-18
T cell exhaustion	PD1 (PDCD1)	0.35	5.56E-12
	CTLA4	0.29	1.22E-08
	LAG3	0.37	1.97E-13
	HAVCR2	0.58	2.39E-34
	GZMB	0.19	2.33E-04
	TOX	-0.13	1.04E-02
	TIGIT	0.33	7.26E-11

TAM, tumor-associated macrophage; Th, T helper cell; Tfh, Follicular helper T cell; Treg, regulatory T cell

**Fig. 5:**
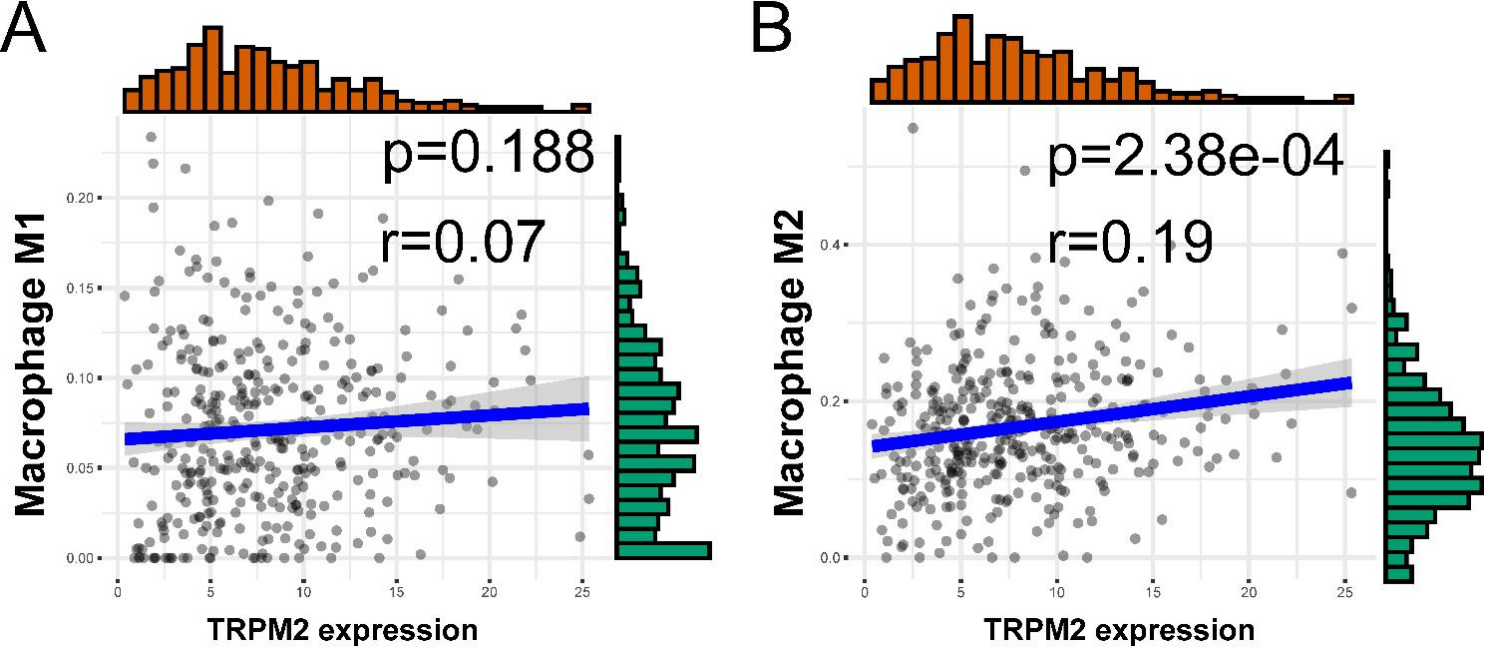
Correlations between TRPM2 and M1/2 macrophage in ovarian cancer depended on TCGA-OV dataset. **(A)** Correlation between TRPM2 expression and M1 macrophage cell. **(B)** Correlation between TRPM2 expression and M2 macrophage cell. (p < 0.05 was considered significant.)

M1 macrophage and M2 macrophage play different roles in tumors. M1 macrophage was often thought to inhibit tumor progression, while M2 macrophage was often considered as a tumor promoter [26]. The result of immune cell markers suggested that TRPM2 expression was positively correlated with M2 macrophage but not correlated with M1 (Table 2). For further verification, we assessed M1 and M2 macrophage infiltration with CIBERSORT and quanTIseq. Consistent with the previous results, there was a stronger correlation between TRPM2 and M2 macrophage than M1 macrophage (Fig. 5A-B) and patients with high expression of TRPM2 also had higher M2 infiltration scores in CIBERSORT analysis (Supplementary Fig. 3B). These results suggested that TRPM2 might play a role in tumor immunity and might promote ovarian cancer progression through participating in the formation of an inhibitory immune microenvironment.

### Correlation between TRPM2 expression and pyroptosis

**Fig. 6:**
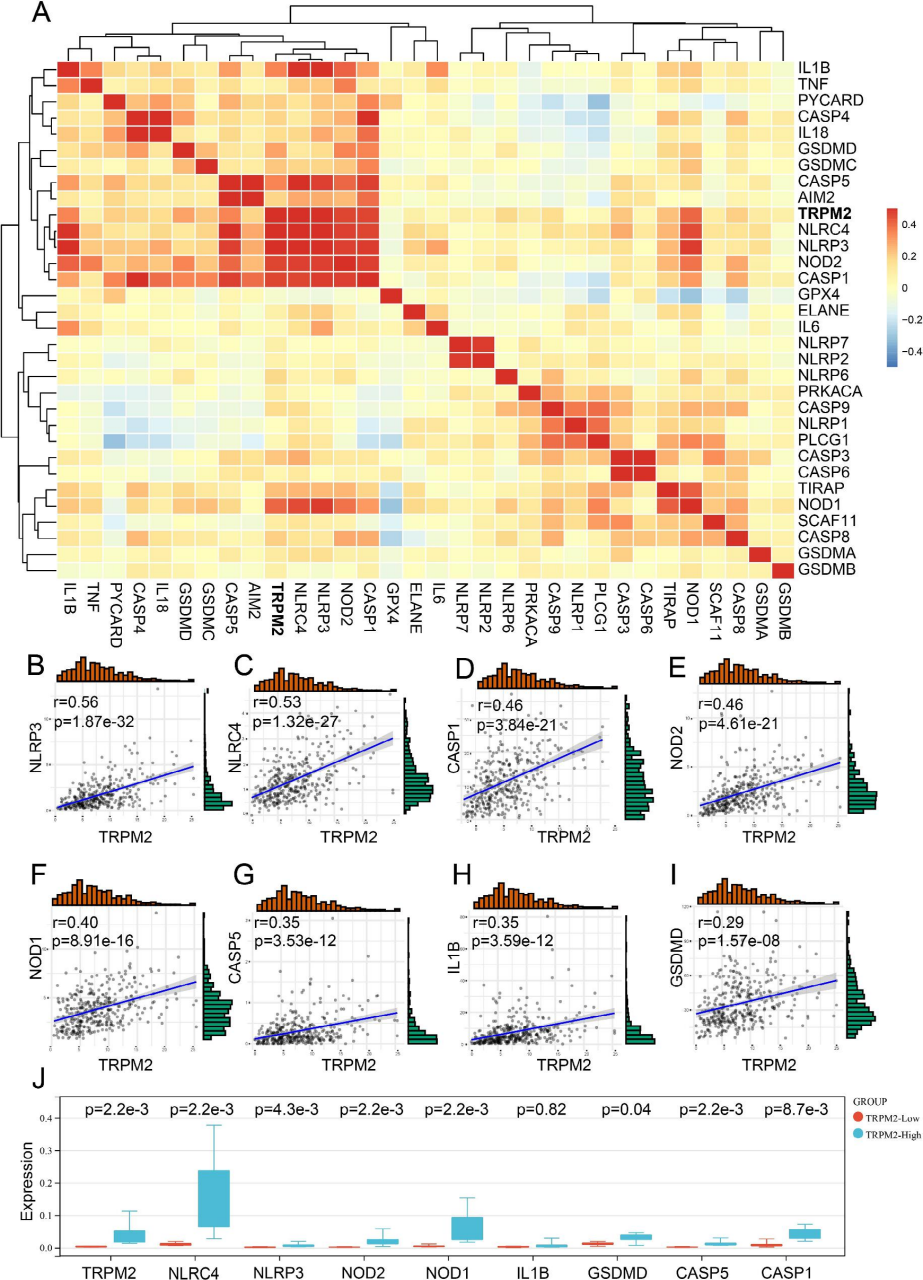
Correlation between TRPM2 expression and pyroptosis-related genes according to TCGA-OV dataset. **(A)** Relationship between TRPM2 and pyroptosis-related genes. **(B-I)** Relationship between TRPM2 and NLRP3 (**B**), NLRC4 (**C**), CASP1 (**D**), NOD2 (**E**), NOD1 (**F**), CASP5 (**G**), IL-1B (**H**), GSDMD (**I**). (**J**) Real-time quantitative PCR result of TRPM2 and pyroptosis-related genes (expression was normalized to GAPDH expression) in clinical samples. N = 5, Student’s t-test. (p < 0.05 was considered significant.)

Previous studies have shown that TRPM2 could activate the NLRP3 inflammasome and induce pyroptosis [27, 28]. Our result also showed high TRPM2 correlated to the acute inflammatory response(Fig. 3A) and innate immune cells (Fig. 4G-I), which were closely associated with pyroptosis [29]. Therefore, we intended to explore the relationship between TRPM2 and pyroptosis-related genes (Fig. 6A, Supplementary Fig. 4A). The result showed that TRPM2 was significantly positively correlated with NLRP3, NLRC4, CASP1, NOD2, NOD1, CASP5, IL1B, and GSDMD in TCGA (Fig. 6B-I). Positive correlations were also observed between TRPM2 and NLRC4, NOD2, CASP1, AIM2, PYCARD, IL18, NLRP3, NLRP1, TNF, and IL1B in GSE17260 (Supplementary Fig. 4B-K). We examined the relationship between TRPM2 and pyroptosis in ovarian cancer by real-time quantitative PCR. The results showed that patients with high TRPM2 expression had both higher NLRC, NLRP3, NOD2, NOD1, GSDMD, CASP5 and CASP1 (Fig. 6J), which was consistent with our results by bioinformatics analysis. The results suggested that high expression of TRPM2 may be positively correlated with pyroptosis pathways in the immune microenvironment.

## Discussion

Ovarian cancer (OC) is the most lethal gynecological malignancy in the world with most patients diagnosed at an advanced stage [1], of which the survival rates remain unsatisfactory for drug resistance and immunosuppressive environment [3]. New therapeutic targets are crucial to improving the survival rate for ovarian cancer patients. Through a series of bioinformatics analyses based on the publicly accessible database, we explored the mRNA expression of TRPM2 in pan-cancer and corresponding normal tissues, and the correlation between TRPM2 expression and survival prognosis and immune microenvironment. Our results showed that TRPM2 was upregulated in most tumors containing ovarian cancer, and increased TRPM2 expression was correlated with poor OS and PFI in patients with OC. Additionally, upregulated TRPM2 expression was positively associated with various immune cell infiltration and immune checkpoints expression. Besides, a close correlation was observed in TRPM2 expression and M2 macrophage, pyroptosis-related genes. Our study demonstrated that TRPM2 was a hazard factor for OC patients which might result from immune regulation, and could be a potential prognosis-predict biomarker for ovarian cancer patients.

TRPM2 is a member of the TRPM ion channel subfamily. Previous reports have proved that TRPM2 promoted tumor progression via multiple mechanisms, such as drug resistance, autophagy, and immunosuppression [14]. Similar results were observed in previous reports, suggesting TRPM2 might act as a risk factor in various tumors through some kind of universal effect [5, 14]. It was reported that TRPM2 sustained cell viability, restore mitochondrial function, reduce reactive oxygen species (ROS) in neuroblastoma, and lead to NLRP3 inflammasome activation [18, 30]. TRPM2 was also proved to preserve gastric cancer cell survival via the JNK-signaling pathway [23]. In this study, we observed abnormal expression of TRPM2 expression in most tumors and survival correlation in some tumors containing ovarian cancer.

The tumor microenvironment is responsible for tumorigenesis and progression, and affects the efficiency of immunotherapy to a certain extent [31]. Adaptive immune cells, including T cells and B cells, are thought to be tumor-killing effector cells, while multiple innate immune cells are thought to play an immunomodulatory function in the tumor microenvironment [32]. Previous studies have proved that TRPM2 existed on monocytes, macrophages, lymphocytes, and neutrophils [11, 12]. In our study, we utilized the GSEA method to analyze the biological process and KEGG pathways. The outcomes demonstrated that high TRPM2 expression was closely related to immune-related pathways such as adaptive immune response, neutrophil mediated immunity, Th1 and Th2 cell differentiation, Th17 cell differentiation, and Natural killer cell mediated cytotoxicity. Additionally, we found that TRPM2 had a positive correlation with multiple immune cells including B cell, T cell, macrophage, neutrophil, and myeloid dendritic cell in OC. We observed that TRPM2 had a stronger correlation with innate immune cells than with adaptive immune cells. In the subsequent correlation analysis of immune cell markers, we also observed a stronger correlation between TRPM2 and innate cell markers and immune regulatory cell markers such as CD86, CSF1R for monocyte, CD11b for neutrophil, CD11c for dendritic cell, and FOXP3 for Treg. The results indicated TRPM2 might mainly participate in immune regulation instead of the direct immune killing effect. Besides, we also observed that TRPM2 was positively correlated with the biomarker genes of M2 macrophages, not M1 macrophages. M1 macrophages are usually considered as tumor suppressor cells, while M2 macrophages are thought to be tumor promoter cells with immunosuppression function [26]. Thus, we speculated that TRPM2 might be involved in the process of promoting tumor progression mediated by M2 macrophages. Together, these results indicated that TRPM2 played an essential role in regulating ovarian cancer immune infiltrating cells.

Immunotherapy, especially immune checkpoints inhibitors, has drawn public attention in the field of cancer treatment [31]. For example, anti-programmed cell death-1 (anti-PD-1), anti-programmed death-ligand 1 (anti-PD-L1), or anti-cytotoxic T-lymphocyte associated protein 4 (anti-CTLA4) have been generally used for non-small cell lung cancer (NSCLC), bladder cancer, heck and neck squamous cell cancer, renal cell carcinoma, melanoma, classical Hodgkin lymphoma, and ovarian cancer [1, 33, 34]. Our result illustrated TRPM2 was significantly positively correlated with ICOSLG, TNFRSF18, CTLA4, CD86, TNFRSF4, CD40, TIGIT, LAIR1, CD48, TNFRSF14, CD27, LGALS9, HAVCR2, KIR3DL1, TMIGD2, CD40LG, ICOS, IDO2, IDO1, PDCD1LG2, and CD80 in OC. This result might explain the high level of immune cell infiltration but the low survival rate of OC patients with high TRPM2 expression. Among the immune checkpoints above, TIGIT is a promising new immunotherapy target, upregulated in activated T cells, natural killer cells, and regulatory T cells [35]. The TIGIT inhibitor tiragolumab has shown effects in multiple tumors [36]. Our study showed that increased TRPM2 was positively correlated to TIGIT, suggesting the potential function of TRPM2 in TIGIT related pathways. Based on the results above, we hope our study on TRPM2 could be helpful for immunotherapy in OC in the future.

Besides, previous reports suggest that TRPM2 can mediate inflammasome-dependent pyroptosis by activating ROS-dependent NLRP3 [27, 28]. Our results also showed that the correlation analysis showed a strong positive correlation between TRPM2 and pyrolysis-related genes including NLRP3, NLRC4, NOD2, NOD1, CASP1, CASP5, IL1B, and GSDMD. Real-time quantitative PCR results validated that patients with high TRPM2 expression had both higher NLRC, NLRP3, NOD2, NOD1, GSDMD, CASP5 and CASP1 (Fig. 6J), which was consistent with our results by bioinformatics analysis. Pyroptosis, often triggered by perturbations of extracellular or intracellular homeostasis related to innate immunity, has been proved to play a dual role in promoting and inhibiting tumor progression in multiple tumors [37, 38]. Combined with the correlation between TRPM2 and innate immunity, we speculated that TRPM2 might be involved in the innate immune-related pyroptosis pathway in OC, resulting in the poor survival of OC patients.

It should be emphasized that there are some limitations to our study. Our study is based on data analysis to mine potential data and functional biomolecules, providing a potential study direction for future research. Experimental studies are needed to verify our results based on designing PCR, Western blotting, and immunohistochemistry tests. More clinical data is needed to prove the relationship between TRPM2 and survival.

## Conclusion

In conclusion, our result found that TRPM2 was upregulated in OC, and high expression of TRMR2 was related to poor prognosis in OC patients. In addition, TRPM2 was mainly related to immune pathways, with a positive correlation with immune cells especially innate immune cells, immune checkpoints, and pyroptosis. It may regard as a new prognostic predictor for OC patients and could be a potential therapeutic target based on further research.

## Materials and methods

### Data collection

The tumor mRNA and clinical data were obtained from The Cancer Genome Atlas (TCGA), a database containing more than 20,000 primary samples and non-cancer samples from 33 types of cancer (https://cancergenome.nih.gov/) [39]. Normal tissue mRNA data was supplemented to normal tissue data in TCGA based on Genotype-Tissue Expression (GTEx) (https://commonfund.nih.gov/GTEx/) [40]. Ovarian cancer dataset GSE17260 was used to verify the analysis result of TCGA-OV according to the GEO database (https://www.ncbi.nlm.nih.gov/geo/).

### Relationship between TRPM2 expression and prognosis value in pan-cancer and ovarian cancer

The correlation between TRPM2 expression and prognosis value including overall survival (OS), disease-specific survival (DSS), disease-free interval (DFI), and progression-free interval (PFI) in pan-cancer was visualized with forest plots. The hazard ratio (HR) and 95% confidence intervals were estimated by univariate survival analysis.

The relationship between TRPM2 expression and clinical characteristics containing age, TNM stage, grade, and race in ovarian cancer was calculated by univariate and multivariate analysis based on Biomedical Informatics Institute (http://bioinfo.henu.edu.cn/DatabaseList.jsp) [41]. Additionally, the correlation between TRPM2 and patient’s prognosis including OS, DSS, 5-year OS, 5-year DSS was visualized with Kaplan-Meir curves according to DriverDBv3 (http://driverdb.tms.cmu.edu.tw/) [25] and PrognoScan (http://dna00.bio.kyutech.ac.jp/PrognoScan/index.html) [24].

### Immunohistochemistry and immunofluorescence

The sections of normal and tumor tissues were fixed overnight in 4% PFA prior to paraffin wax professing and embedding. Tissue sections were cut at 4µm size. Endogenous peroxidase was blocked with 0.3% hydrogen peroxide for 30 minutes in adjacent sections. Antigen was retrieved by heating at 100°C for 30 minutes. Slides were then incubated with the anti-TRPM2 (ZENBIO, China) for 1 hour. A labeled streptavidin-biotin system with a horse-radish peroxidase label was used to detect the primary antibodies and visualized by incubation with 3,3’-diaminobenzidine chromogen and hydrogen peroxide substrate for 10 min. The slides were then counterstained with hematoxylin and mounted in dibutyl phthalate xylene.

### Functional enrichment analysis

The RNA expression data obtained from TGCA-OV and GSE17260 datasets were normalized by the log_2_(X + 1) algorithm and then the fold change value was calculated by the R package “limma” according to TRPM2 expression. Functional enrichment analysis was estimated by the gene set enrichment analysis (GSEA) algorithm. We perform the GO function enrichment analysis, KEGG pathways enrichment analysis depending on the WEB-based Gene SeT AnaLysis Toolkit (WebGestalt, http://www.webgestalt.org/) according to log_2_FC value [42].

### Correlation analysis of TRPM2 expression with immune characteristics

We assessed the relationship between TRPM2 expression and ImmuneScore, StromalScore, and ESTIMATEScore by ESTIMATE [43]. We utilized the RNA-seq expression profile to estimate infiltration of immune cells covering B cells, CD8^+^ T cells, CD4^+^ T cells, macrophage, neutrophil, and myeloid dendritic cells based on TIMR2.0 (http://timer.comp-genomics.org/) [44]. M1 and M2 macrophage infiltration were assessed with CIBERSORT [45]. Subsequently, correlation analysis was performed to evaluate the relationship between TRPM2 expression and immune infiltration, immune checkpoints, and immune cell markers. The data were visualized by the R package “ggcorrplot”.

### Correlation analysis TRPM2 expression with pyroptosis

The correlation analyses between TRPM2 expression and pyroptosis-related genes were performed with the R-package “psych” and visualized by the R-package “pheatmap” in OC based on TCGA and GSE17260 databases.

### RNA isolation and quantitative RT-PCR

Total RNA was extracted from tissues or cells using Trizol reagent (Invitrogen, Carlsbad, CA, USA) according to the manufacturer’s instructions. The mRNA levels of TRPM2 and pyroptosis-related genes were determined by quantitative RT-PCR using the SYBR Green (Thermo Fisher Scientific, Rockford, IL, USA), with GAPDH as an internal control. The primers are listed in Supplementary Table 1. All reactions were conducted using the following cycling parameters, 95℃ for 10 min, followed by 40 cycles of 95℃ for 15s, and 60℃ for 45s. Verification of specific product amplification was determined by dissociation curve analysis. The comparative Ct method was used for the quantification of the transcripts. The fold-change for target genes normalized by internal control was determined by the formula 2-△CT.

### Statistical analysis

In survival analysis, the HRs and p-values were estimated by univariate Cox regression analysis. Kaplan-Meier curves was used to analyze the survival of patients stratified based on different levels of TRMR2 expression. All correlation analyses were adopted by coefficient Pearson. The level of significance was set as p < 0.05.

### Electronic supplementary material

Below is the link to the electronic supplementary material.

**Supplementary Material 1**
