# Supplementary material for: Identification of TRPM2 as a prognostic factor correlated with immune infiltration in ovarian cancer
Source: J Ovarian Res. 2023 Aug 22;16:169. doi: 10.1186/s13048-023-01225-y (PMC10463424; doi:10.1186/s13048-023-01225-y)
Supplement: Supplementary file 1 — Supplementary Material 1 [file 13048_2023_1225_MOESM1_ESM.docx]

**Supplementary Figure 1**





**Supplementary Fig. 1.** Survival analysis of TRPM2 in human pan-cancer. (**A-C)** Disease-specific survival (DSS) **(A)**, progression-free survival (PFI) **(B)**, and disease-free interval (DFI) **(C)** analysis of TRPM2 in human pan-cancer. (p<0.05 was considered significant.)

**Supplementary Figure 2**





**Supplementary Fig. 2.** KEGG pathways of TRPM2 in TCGA and GSE17260 dataset. (**A)** KEGG pathways in TCGA-OV. (**B)** KEGG pathways of immune-related in TCGA-OV. **(C)** KEGG pathways in GSE17260. **d** KEGG pathways of immune-related in GSE17260.

**Supplementary Figure 3**


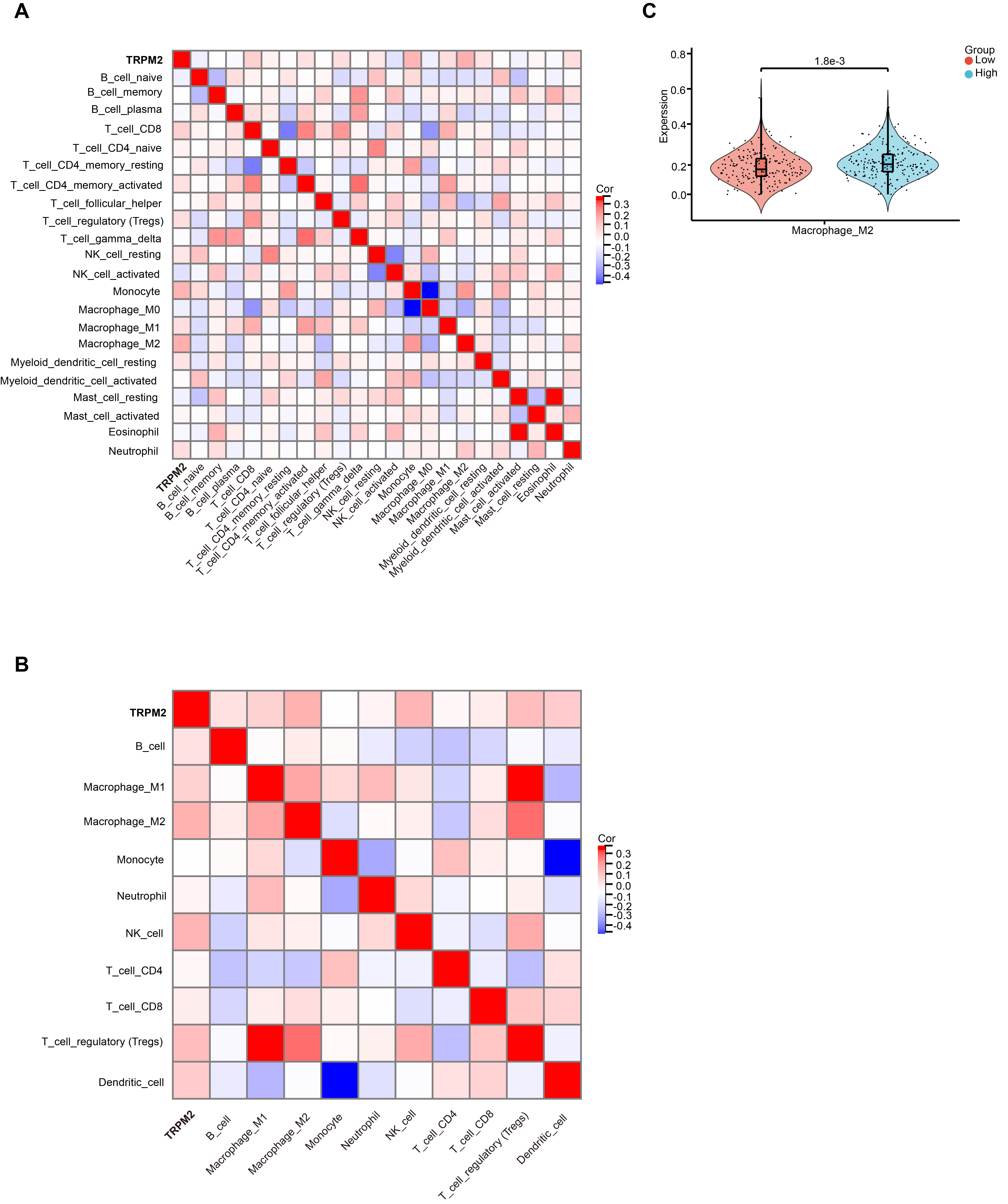


**Supplementary Fig. 3.** Correlation between TRPM2 expression and immune cell infiltration. (**A)** Correlation between TRPM2 expression and immune cell infiltration analysed by Cibersort. (**B)** The differences in M2 macrophages between groups with high- and low-expression of TRPM2 (**C**) Correlation between TRPM2 expression and immune cell infiltration analysed by quanTIseq.

**Supplementary Figure 4**





**Supplementary Fig. 4.** Correlation between TRPM2 expression and pyroptosis-related genes according to GSE17260 dataset. (**A)** Relationship between TRPM2 and pyroptosis-related genes. (**B-K)** Relationship between TRPM2 and NLRP3 (**B**), NOD2 (**C**), CASP1 (**D**), AIM2 (**E**), PYCARD (**F**), IL18 (**G**), NLRP3 (**H**), NLRP1(**I**), TNF(**J**), IL1B (**K**). (p<0.05 was considered significant.)

**Supplementary Table**

Supplementary Table1. Primer sequence used in the study.

| gene | F | R |
| --- | --- | --- |
| NLRP3 | GATCTTCGCTGCGATCAACAG | CGTGCATTATCTGAACCCCAC |
| NLRC4 | TGCCCAGAAATCGAAGCCC | GGCACCAAACTGCCGTATG |
| CASP1 | TTTCCGCAAGGTTCGATTTTC | GGCATCTGCGCTCTACCATC |
| NOD2 | TGGTTCAGCCTCTCACGATGA | CAGGACACTCTCGAAGCCTT |
| NOD1 | ACTGAAAAGCAATCGGGAACTT | CACACACAATCTCCGCATCTT |
| CASP5 | TTCAACACCACATAACGTGTCC | GTCAAGGTTGCTCGTTCTATGG |
| IL1B | AGCTACGAATCTCCGACCAC | CGTTATCCCATGTGTCGAAGAA |
| GSDMD | GTGTGTCAACCTGTCTATCAAGG | CATGGCATCGTAGAAGTGGAAG |
| TRPM2 | TTCGTGGATTCCTGAAAACATCA | CCAGCATCAGACAGTTTGGAAC |
